# Sexual and gender minority stigma and motivating beliefs among the general public in Rwanda

**DOI:** 10.3389/fsoc.2025.1597223

**Published:** 2025-10-01

**Authors:** Gustave Muhire, Ann Chereen Karanja, Odile Habimana, Everest Turatsinze, Deborah Kansiime, Louange Gutabarwa Twahirwa, Alain Favina, Ritah Mukashyaka, Egide Niyotwagira, Aflodis Kagaba, Emmy Kageha Igonya, Emmanuel Otukpa, Kristefer Stojanovski

**Affiliations:** ^1^Health Development Initiative (HDI), Kigali, Rwanda; ^2^Health and Wellbeing Unit, African Population and Health Research Center, Nairobi, Kenya; ^3^Department of Social, Behavioral, and Population Sciences, Celia Scott Weatherhead School of Public Health & Tropical Medicine, Tulane University, New Orleans, LA, United States

**Keywords:** discrimination, stigma, perceptions, sexual and gender minorities, Rwanda

## Abstract

**Background:**

Sexual and gender minorities (LGBTQ+) individuals in Rwanda face significant stigma and discrimination, driven by stigmatizing sociocultural and religious norms. This study investigates public perceptions toward sexual and gender minorities (LGBTQ+) in Rwanda, focusing on exploring non-discrimination beliefs, acceptance levels, and views on whether LGBTQ+ identities are innate.

**Methodology:**

A cross-sectional study was conducted across six districts in Rwanda, with 1,254 non-LGBTQ+ participants using convenience and snowball sampling. Descriptive statistics, ANOVA, and multivariable linear regression analyses were performed to assess associations between non-discrimination beliefs, acceptance, beliefs about being born LGBTQ+, and sociodemographic factors.

**Results:**

The average LGBTQ+ acceptance score was 8.7 out of 15 (Stdev: 3.6). Beliefs that one is born LGBTQ+ had an average score of 6.0 out of 10 [Sdev=2.2]. Regarding beliefs that one should not discriminate against LGBTQ+ the average score was 28.0 [Stdev: 8 out of 39.2]. Every one-point increase in people's beliefs about not discriminating against LGBTQ+, their acceptance of LGBTQ+ people increases by 0.25 points [95% CI (0.23, 0.27)]. As people's beliefs about not discriminating against LGBTQ+ increased by one point their belief that LGBTQ+ are born as such increases by 0.11 points [95% CI (0.10,0.12)].

**Conclusion:**

Most Rwandans sampled have non-discriminatory attitudes, however the acceptance of LGBTQ+ persons and beliefs that being LGBTQ+ is innate remains low. As non-discriminatory beliefs improve so does acceptance of LGBTQ+ and the belief that it is an innate identity. The findings suggest that educational and sensitivity efforts might be worth exploring as they could potentially improve attitudes toward perceptions and acceptance toward LGBTQ+ individuals, but experimental research would be needed to test this possibility.

## 1 Introduction

Rwanda stands out in the African context for not criminalizing same-sex relationships and for having adopted several international human rights instruments protecting sexual and gender minorities (LGBTQ+). However, despite this progressive legal environment, LGBTQ+ individuals in Rwanda continue to face stigma, discrimination, and marginalization in various sectors, including healthcare, education, the workplace, and within their families. Recent studies conducted among key populations, such as men who have sex with men (MSM), transgender sex workers (TSW), and lesbians and bisexual women, highlight persistent experiences of mistreatment, external stigma, and rejection in both public and private settings (Paszat, 2022; Isano et al., 2023; Moreland et al., 2019). These lived experiences expose a gap between Rwanda's formal legal protections and informal social norms, indicating that acceptance of LGBTQ+ individuals remains limited.

Understanding public perceptions and the underlying belief systems that drive stigma and discrimination is therefore critical. Beliefs about whether LGBTQ+ individuals are “born this way,” as well as general attitudes toward their rights and inclusion, play a significant role in shaping behaviors and policy support (Ayhan et al., 2020). Theoretical concepts such as othering—the social construction of certain groups as fundamentally different and inferior—help explain the mechanisms through which stigma and exclusion are maintained (Goffman, 1959). In Rwanda, as in many African contexts, cultural and religious narratives often frame LGBTQ+ identities as un-African or immoral, reinforcing social distance and discrimination (Cheney, 2012; Bills and Hayes, 2020). These ideologies intersect with gender norms and patriarchal structures, where deviation from prescribed roles is penalized. For example, mothers of LGBTQ+ children are often blamed, reflecting gendered ideologies that associate child development solely with maternal influence (Chess, 1982; Colker, 2015; Toews et al., 2019; Sommerfeld, 1989; 'T Hart, 2024; Goodhart, 2021; Kolker, 2021).

Comparative insights from other countries further contextualize Rwanda's situation. In the U.S., despite broader LGBTQ+ rights, transgender individuals face disproportionate stigma, showing how identity-specific attitudes matter (Lewis et al., 2017). In South Africa, despite progressive laws widespread homophobia persists, revealing how legal advances do not always translate into social acceptance (Statista, 2024; Mahomed and Trangoš, 2016). In several African nations, religious and cultural beliefs continue to drive political resistance to LGBTQ+ inclusion, a pattern mirrored to some extent in Rwanda's social attitudes (Bills and Hayes, 2020; Alozie et al., 2017).

Given this context, this study investigates how anti-discrimination beliefs, acceptance of LGBTQ+ individuals, and essentialist beliefs (such as being “born this way”) are interrelated in shaping public attitudes. By focusing on these constructs, the study aims to inform efforts that promote genuine inclusion and challenge stigmatizing beliefs in Rwanda.

## 2 Material and methods

### 2.1 Study setting

**Figure 1:**
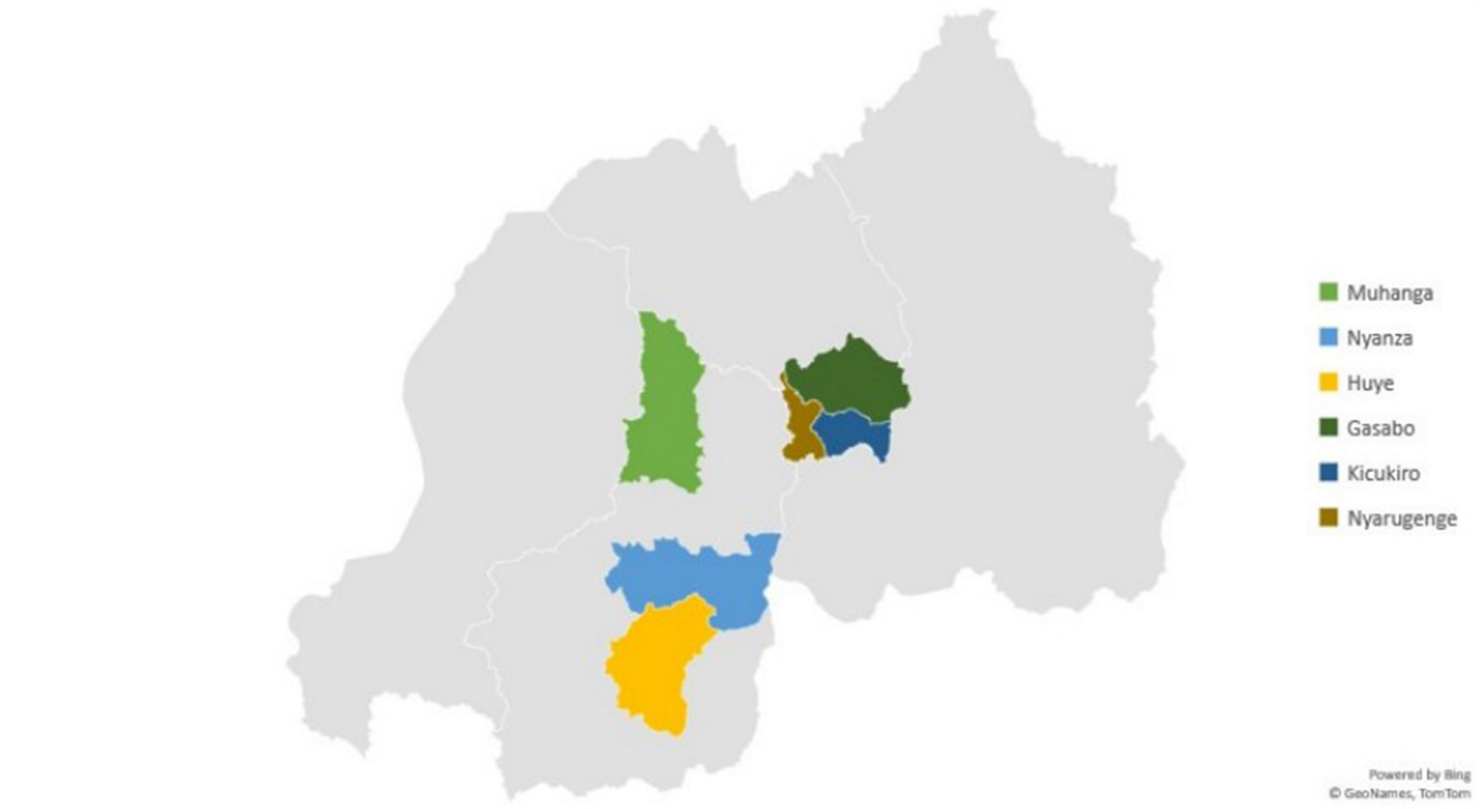
Districts where study was conducted.

The study was conducted across six districts in Rwanda: Nyarugenge, Gasabo, and Kicukiro in Kigali, as well as Huye, Nyanza, and Muhanga in the Southern Province from May to December 2021 (Figure 1). These districts were strategically selected to provide a comprehensive understanding of public perceptions toward sexual and gender minorities in Rwanda. The Southern Province, particularly Nyanza, which hosts the King's palace, and Huye, home to the Rwanda Ethnographic Museum and the University of Rwanda, is considered the cultural heartland of the country, making it a crucial area for understanding community beliefs, attitudes, and norms.

### 2.2 Study design

We conducted a sequential cross-sectional mixed methods study to study the acceptance level of LGBTQ+ people among the public. This analysis is based on a quantitative survey of the general public. The quantitative surveys aimed at assessing the public perception of LGBTQ+ people by measuring attitudes regarding LGBTQ+ people and participant demographics such as age, religion, region, and occupational status. Further details about the study design and methods have been published elsewhere (Stojanovski et al., 2024).

### 2.3 Study participants

The study employed convenience and purposive sampling to choose the participants. We recruited 1,254 participants from the general population, including religious leaders, local authorities, and business owners. We recruited from markets, shops, bus terminals and churches. A community mobilizer helped us to sample key informants and places where to find people who can provide rich insights to the survey. The inclusion criteria were as follows: (1) had to be 18 years or above, (2) able to provide informed consent and (3) able to speak in Kinyarwanda. People unable to provide consent and people who lived in the area for < 6 months were excluded.

### 2.4 Data collection

The study aimed to assess societal attitudes and perceptions toward the LGBT community in Rwanda. Questions were adapted from a previous survey conducted in South Africa on General public perception of LGBTQ people. Questions adapted included: (1) I accept gay people as part of society and (2) Gays and Lesbians deserve the same human rights as heterosexuals to make it less convoluted for the Rwandan context (The Other Foundation, 2016). The questionnaire gathered questions on respondents' demographic characteristics, their views on LGBTQ+ people being born queer, their acceptance level of LGBTQ+ people, and their attitudes toward LGBTQ+ people.

The survey was conducted by well-trained research assistants using real-time data capture via Survey CTO. Research assistants underwent comprehensive training on bias awareness, data collection, human subjects, and ethics and followed standardized data collection procedures. The principal investigator and co-investigators closely monitored data collection and implemented spot-checks on 5% of the sample to verify data accuracy. The research team held weekly meetings to address quality and performance issues.

### 2.5 Variables and measures

#### 2.5.1 Outcome variables

We had two outcomes. The first was a measure of LGBTQ+ acceptance. It was measured using three questions which asked: “Generally, I feel (1) positive toward LGBT people, (2) I believe LGBT should be treated like any other person under the law, and (3) I support LGBT rights.” Each one had a Likert response option ranging from strongly agree (1) to disagree (5) strongly. We reversed the values such that higher scores meant more acceptance. The alpha was 0.80 with a possible range of 3–15.

The second outcome was beliefs about whether LGBTQ+ were born that way. This was measured by two questions: (1) one is born homosexual, straight or bisexual and (2) one is born transgender. The response options were a Likert scale ranging from strongly agree (1) to strongly disagree (5). We reversed the scores such that higher scores meant they strongly agreed that LGBTQ+ were born as such. The alpha was 0.61, with a possible range of 2–10.

#### 2.5.2 Main independent variables

The primary predictor was beliefs about not discriminating against LGBTQ+, which was measured with an eight-part question that asked, “It is important not to discriminate against LBGTQ+ in…” (1) the workplace, (2) education institutions, (3) places of worship, (4) homes, (5) communities, (6) health facilities, (7) public spaces, and (8) social welfare/protection. Each one had a Likert response option ranging from strongly agree (1) to strongly disagree (5). We reverse-scored the measure such that higher scores mean more positive responses (less discriminatory beliefs). The alpha was 0.93, with a possible range of 10–39.

#### 2.5.3 Covariates

The sociodemographic characteristics were as follows: gender measured dichotomously as male or female, age, which was a continuous variable in years, geographic residence dichotomized as urban or rural, level of education categorized into pre-primary, primary, secondary, vocational or bachelor or higher, employment status categorized as unemployed, employed or self-employed, and religion which was categorized as Christian, Islam and traditional/other.

### 2.6 Statistical analyses

The data was first cleaned to check for any missing values and inconsistencies ensuring the validity and reliability of the data set. The data was then transferred to Stata 14.0 for analysis (Statacorp, 2024). We used descriptive statistics to summarize the quantitative data on respondents' views on LGBTQ+ people being born as queer, their acceptance level of LGBTQ+ people and their discrimination beliefs toward LGBTQ+ people providing means, standard deviations the minimum and maximum scores. We also examined sociodemographic characteristics using frequencies and percentages. We conducted bivariate analyses using independent *t*-tests or one-way ANOVAs to examine variation in means of the outcomes by categorical variables. We report means, standard deviations, t or f statistics, and their respective *p*-values. We conducted unadjusted linear regression analyses to examine views about LGBTQ+ acceptance and being born LGBTQ+ by the main predictor of non-discriminatory attitudes and respective socio-demographics. Finally, we conducted adjusted linear regression models of the two outcomes separately. We report unadjusted and adjusted beta coefficients and their 95% confidence intervals and test statistics.

### 2.7 Ethical considerations

The APHRC Internal Ethics Review Committee first reviewed and approved the study protocol. The protocol was then submitted to the Rwanda National Ethics Committee and was approved (No. 117/RNEC/2021). All persons who agreed to participate in the study gave informed consent. The study team anonymized all data, assigning participants unique identifiers to protect their identities.

## 3 Results

### 3.1 Sociodemographic

The sample consisted of 1,254 participants, with 61. The sample was predominantly urban (79.5%) and relatively well-educated, with 40% having vocational or higher education 0.0% (*n* = 762) identifying as male and 38.0% (*n* = 479) as female. Half the sample was 30 years of age (Stdev = 8.9). The majority resided in urban areas (79.5%, *n* = 997). Educational attainment varied across the sample: 11.5% (*n* = 145) had a pre-primary education, 24.4% (*n* = 307) had completed primary education, 23.4% (*n* = 294) had secondary education, 32.1% (*n* = 403) had vocational training, and 8.3% (*n* = 105) held a bachelor's degree or higher. Regarding religious affiliation, 79.4% (*n* = 1,000) identified as Christian, 9.6% (*n* = 121) as Muslim, and 10.6% (*n* = 133) as practicing other religions. In terms of employment status, 34.6% (*n* = 434) were unemployed, 36.1% (*n* = 453) were employed, and 29.2% (*n* = 367) were self-employed.

### 3.2 Views about LGBTQ+ acceptance, being born LGBTQ+, and non-discrimination

The average score on the LGBTQ+ acceptance variable was 8.7 (Stdev: 3.6, Range: 3–15). The average score on whether participants believed that LGBTQ+ people are born queer was 6.0 (Stdev: 2.2, Range: 2–10). Regarding beliefs that one should not discriminate against LGBTQ+ the average score was 28.0 (Stdev: 8 out of 39.2, Range: 10–39).

Results indicated that beliefs about LGBTQ+ people significantly varied by age, education, employment, and beliefs about non-discrimination. For education, ANOVA tests showed that as levels of education increased the average acceptance scores increased, except it was only significant for those with vocational levels (mean = 9.4), as compared to pre-primary (mean = 7.95, *p* < 0.004) and primary education (mean = 8.02, *p* < 0.000). For employment status, the ANOVA test showed a lower acceptance level of LGBTQ+ people among people who are self-employed (mean = 8.08) compared to those unemployed (mean = 9.05, *p* < 0.001) and employed (mean = 8.92, *p* < 0.005). No other variables were significant in the independent *t*-tests or ANOVAs (Table 1).

**Table 1:** Sociodemographic and associations with discrimination, understanding and acceptance of LGBTQ+ people in Rwanda (*n* = 1,254).

**Variable**		**LGBTQ**+ **acceptance**				**Born LGBTQ**+				**Discrimination beliefs**			
	***N*** **(%)**	***N*** **(%)**	**Mean**	**Stdev**	**T/F-statistic**	***N***	**Mean**	**Stdev**	**T-statistic**	***N***	**Mean**	**Stdev**	**T-statistic**
**Sex**					−1.07				−0.114				−1.47
Male	762 (60.8%)	711 (61.8%)	8.59	3.68		762 (60.8%)	5.93	2.18		762 (60.8%)	27.7	8.4	
Female	479 (38.2%)	440 (38.2%)	8.83	3.460		479 (38.2%)	5.94	2.09		479 (38.2%)	28.4	8.1	
**Educational level**					7.74				12.36				7.17
Pre-primary	145 (11.6%)	131 (11.3%)	7.95	3.58		145 (11.6%)	5.64	2.03		145 (11.6%)	26.5	8.4	
Primary	307 (24.5%)	291 (25.0%)	8.02	3.66		307 (24.5%)	5.36	2.15		307 (24.5%)	26.6	8.5	
Secondary	294 (23.4%)	270 (23.2%)	8.81	3.43		294 (23.4%)	5.97^*^	2.07		294 (23.4%)	27.8	7.9	
Vocational	403 (32.1%)	375 (32.2%)	9.37^*^	3.60		403 (32.1%)	6.42^*^	2.15		403 (32.1%)	29.6^*^	8.1	
Bachelor or higher	105 (8.4%)	96 (8.3%)	9.08	3.6		105 (8.4%)	6.27^*^	2.13		105 (8.4%)	28.3	8.1	
**Employment status**					7.88				10.15				1.5
Unemployed	434 (34.6%)	400 (34.4%)	9.05	3.56		434 (34.6%)	5.88	2.14		434 (34.6%)	28.3	8.0	
Employed	453 (36.1%)	422 (36.3%)	8.92	3.59		453 (36.1%)	6.28	2.18		453 (36.1%)	28.1	8.4	
Self employed	367 (29.3%)	341 (29.3%)	8.08^*^	3.65		367 (29.3%)	5.62	2.09		367 (29.3%)	27.4	8.3	
**Religion**					0.11				0.73				0.04
Christianity	1,000 (79.7%)	934 (80.3%)	8.73	3.56		1,000 (79.7%)	5.99	2.15		1,000 (79.7%)	28.0	8.2	
Islam	121 (9.7%)	112 (9.6%)	8.80	3.81		121 (9.7%)	5.83	2.05		121 (9.7%)	27.9	8.2	
Other/no religion	133 (10.6%)	117 (10.1%)	8.59	3.84		133 (10.6%)	5.78	2.29		133 (10.6%)	28.1	8.4	
**Location**													0.457
Urban	997 (79.5%)	916 (78.8%)	8.76	3.60		997 (79.5%)	6.00	2.12		997 (79.5%)	28.0	8.2	
Rural	257 (20.5%)	247 (21.2%)	8.57	3.68		257 (20.5%)	5.75	2.28		257 (20.5%)	27.8	8.3	

**Acceptance**

^*^Significant for those having a vocational level of education compared to those having a preprimary level of education p < 0.004 and primary level *p* < 0.000.

^*^Significant for those who are self-employed compared to the unemployed *p* < 0.001 and employed p < 0.005.

**Born LGBTQ+**

^*^Significant for those having a secondary level of education compared to those having a primary level of education *p* < 0.013.

^*^Significant for those having a vocational level of education compared to pre-primary level of education p < 0.007 and primary level of education *p* < 0.000.

^*^Significant for those having a bachelor or higher level of education compared to primary level of education *p* < 0.005.

^*^Significant for those who are employed compared to the unemployed *p* < 0.021.

^*^Significant for those who are self-employed compared to the employed *p* < 0.000.

**Discrimination beliefs**

^*^Significant for those with a vocational level of education compared to the pre-primary level *p* < 0.005, primary level *p* < 0.000 and secondary level *p* < 0.082.

Results for beliefs about LGBTQ+ being born queer varied by certain sociodemographic. Results also showed that there is a higher belief that a person is born as a LGBTQ+ among employed people (mean = 6.28), as compared to unemployed people (mean = 5.88, *p* < 0.021) and self-employed (mean = 5.62, *p* < 0.000). Participants with secondary education, as compared to primary, had higher scores (5.97 vs. 5.36, *p* < 0.013). Persons with vocational level had an average score of 6.4 compared to 5.6 for those with pre-primary education and 5.4 for those with primary education (*p* < 0.007 & *p* < 0.000, respectively). Those with a bachelor's degree or higher had a statistically higher average score of 6.3 compared to those with primary education (*p* < 0.005). No other variables were associated in the chi-square tests (Table 1).

Results indicated that beliefs that LGBTQ+ people should not be discriminated against varied only by the level of education. There was no significant association between gender, religion, or location and non-discrimination beliefs. Participants with a vocational level of education had an average score of 29.6 as compared to 26.5 for those with pre-primary (*p* < 0.005), 26.6 for those with primary education (*p* < 0.000), and 27.8 for those with secondary education (*p* < 0.082).

### 3.3 Unadjusted results of LGBTQ+ acceptance and being born LGBTQ+

In unadjusted linear models of LGBTQ+ acceptance, results showed that beliefs about non-discrimination, age, education, and employment status were all significantly associated with LGBTQ+ acceptance. For every one-year increase in age, acceptance scores were reduced by 0.07 points [95% CI (−0.09, −0.04)]. For every one-unit increase in non-discrimination attitudes, the acceptance scores of the LGBTQ+ community increased by 0.26 [95% CI (0.12, 0.28)]. People with a secondary, vocational, and bachelor education had higher LGBTQ+ acceptance, and it went up by 0.86 [95% CI (0.11, 1.61)], 1.42 [95% CI (0.70, 2.13)], and 1.13 [95% CI (0.19, 2.07)], respectively. Results also showed that the acceptance level of LGBTQ+ people among people who were self-employed as compared to those unemployed were 0.98 points lower [95% CI (−1.50, −0.46)].

In unadjusted linear models of being born LGBTQ+, results showed that for a 1-year increase in age, beliefs that one is born LGBTQ+ went down by 0.43 [95% CI (−0.06, −0.03)]. As people's beliefs about not discriminating against LGBTQ+ increase by one point, their belief that LGBTQ+ are born as such increases by 0.12 points [95% CI (0.10, 0.13)]. For people with a vocational and bachelor education, the attitude scores toward being born LGBTQ+ were higher by 0.76 [95% CI (0.37, 1.18)] and 0.63 [95% CI (0.10, 1.17)], respectively. Results also indicated that attitudes toward being born LGBTQ+ among employed people compared to the unemployed were 0.40 points higher [95% CI (0.12, 0.68)]. The results showed no association between gender, religion, and location with LGBTQ+ acceptance and attitude toward being born LGBTQ+ (Table 2).

**Table 2:** Unadjusted and adjusted beta coefficients of factors influencing LGBTQ+ acceptance and perceptions of being born LGBTQ+ in the general public.

**Variable**	**LGBTQ acceptance**						**Born LGBTQ**+					
	**Unadjusted B**	**95% (CI)**	**T-statistic**	**Adjusted B**	**95% (CI)**	**T-statistic**	**Unadjusted B**	**95% (CI)**	**T-statistic**	**Adjusted B**	**95% (CI)**	**T-statistic**
**Variable**												
Non-discrimination beliefs	0.26	(0.24, 0.28)^*^	26.35	0.25	(0.23, 0.27)^*^	25.09	0.12	(0.10, 0.13)^*^	17.84	0.11	(0.10, 0.12)^*^	16.80
Age	−0.07	(−0.09, −0.4)^*^	−5.67	−0.03	(−0.05, −0.01)^*^	−3.25	−0.43	(−0.06, −0.03)^*^	−6.51	−0.03	(−0.04, −0.02)^*^	−4.46
**Sex**												
Male	ref			ref	–	–	ref	–	–	ref	–	–
Female	0.23	(−0.19, 0.66)	1.07	0.06	(−0.28, 0.40)	0.35	0.01	(−0.23, 0.26)	0.11	−0.08	(−0.29, 0.15)	−0.64
**Educational level**												
Pre-primary	ref			ref	–	–	ref	–	–	ref	–	–
Primary	0.62	(−0.67, 0.80)	0.17	−0.04	(−0.63, 0.55)	−0.13	−0.29	(−0.70, 0.13)	−1.34	−0.36	(−0.73, 0.02)	−1.86
Secondary	0.86	(0.11, 1.61)^*^	2.26	0.36	(−0.24, 0.96)	1.18	0.33	(−0.09, 0.75)	1.54	0.03	(−0.35, 0.41)	0.16
Vocational	1.42	(0.70, 2.13)^*^	3.91	0.27	(−0.32, 0.85)	0.90	0.76	(0.37, 1.18)^*^	3.78	0.18	(−0.19, 0.55)	0.95
Bachelor or higher	1.13	(0.19,2.07)^*^	2.35	0.44	(−0.32, 1.21)	1.13	0.63	(0.10, 1.17)^*^	2.35	0.20	(−0.28, 0.69)	0.81
**Employment status**												
Unemployed	ref			ref	–	–	ref	–	–	ref	–	–
Employed	−0.13	(−0.62, 0.37)	−0.5	−0.11	(−0.51, 0.30)	−0.52	0.40	(0.12, 0.68)^*^	2.78	0.38	(0.12, 0.64)^*^	2.85
Self employed	−0.98	(−1.50, −0.46)^*^	−3.69	−0.61	(−1.04, −0.19)^*^	−2.83	−0.27	(−0.56, 0.03)	−1.75	−0.01	(−0.28, 0.26)	−0.07
**Religion**												
Christianity	ref			ref	–	–	ref	–	–	ref	–	–
Islam	0.08	(−0.63, 0.79)	0.21	0.03	(−0.54, 0.59)	0.09	−0.15	(−0.56, 0.25)	−0.74	−0.19	(−0.55, 0.17)	−1.03
Other/no religion	−0.14	(−0.83, 0.56)	−0.039	−0.14	(−0.69, 0.41)	−0.49	−0.21	(−0.60, 0.18)	−1.04	−0.20	(−0.55, 0.14)	−1.15
**Location**												
Urban	ref			ref	–	–	ref	–	–	ref	–	–
Rural	−0.18	(−0.69, 0.32)	−0.71	−0.11	(−0.52, 0.30)	−0.52	−0.25	(−0.54, 0.05)	−1.64	−0.10	(−0.36, 0.17)	−0.70

^*^Denotes statistical significance.

### 3.4 Adjusted results of LGBTQ+ acceptance and being born LGBTQ+

In adjusted linear regression models of LGBTQ+ acceptance, results showed that beliefs about non-discrimination, age, and employment status were significantly associated with LGBTQ+ acceptance. As people's beliefs about not discriminating against LGBTQ+ increase by one point, their acceptance of LGBTQ+ people increases by 0.25 points [95% CI (0.23, 0.27)]. Results indicated that for every 1-year increase in age, acceptance of LGBTQ+ people decreased by 0.03 points [95% CI (−0.05, −0.01)]. Self-employed persons, as compared to unemployed, had an LGBTQ+ acceptance score that was 0.61 points lower [95% CI (−1.04, −0.19)]. No other variables were significantly associated with LGBTQ+ acceptance (Table 2).

In adjusted linear regression models of beliefs about LGBTQ+ being born queer, results showed that beliefs about non-discrimination, age, and employment status were all significantly associated with beliefs that LGBTQ+ people are born queer. As people's beliefs about not discriminating against LGBTQ+ increased by one point, their belief that LGBTQ+ is born as such increased by 0.11 points [95% CI (0.10, 0.12)]. As people's age increases, the belief of being born LGBTQ+ decreases by 0.03 points [95% CI (−0.04, −0.02)]. Compared to unemployed people, employed people had 0.38 higher LGBTQ+ are born this way scores [95% CI (0.12, 0.64)]. The results showed no association between gender, religion and location of the respondents with LGBTQ+ acceptance and beliefs that one is born LGBTQ+ (Table 2).

## 4 Discussions

The findings in our study show that even though the general population holds positive beliefs that one should not discriminate against LGBTQ+ people, the population lower levels of LGBTQ+ acceptance and misinformed beliefs about LGBTQ+ being “born this way.” The results also showed that age, employment status, and education level are significantly associated with the community's acceptance toward LGBTQ+ and correct beliefs that people are born LGBTQ+ The study suggests that among this sample of Rwandans, attitudes toward LGBTQ+ individuals reflect both non-discriminatory beliefs and persistent stigma regarding LGBTQ+ people is discriminatory and misinformed, but there are these associations suggest interventions might be worth exploring.

The multidimensionality of LGBTQ+ stigma in Africa is complex. Gender and its social construction play a critical role in stigmatizing LGBTQ+ persons, given the strong hegemonic heteronormative sexual orientation in Africa (Amoah and Gyasi, 2016). There are strong societal expectations on how men and women should act, dress, and interact in Africa. As an example, these norms have led to gay men being stigmatized in society for not adhering to traditional notions of masculinity (Lopang, 2014; Ratele, 2013). Lesbian women have been victims of “corrective” rape to “straighten” their sexual orientation toward heteronormativity (Morrissey, 2013). Anti-LGBTQ+ norms and values are rooted in various issues. First are deep religious convictions and beliefs that make it possible for people and the government to discriminate against LGBTQ+ and others who “challenge” their religious beliefs^1^ (Tarusarira, 2024). Gender in religious contexts also plays a role by depicting man as “God's firstborn” created to dominate the earth and women and as a second thought to comfort the domineering man, hence intolerant of other sexualities (Izugbara, 2004).

Community norms are the set of formal and informal agreements about what is perceived to be “normative” or “unnatural” based on a set of beliefs, values, and perceptions. Community norms are what people believe others think about the issue vs. one's internal attitudes and beliefs. Social referents', individuals with influence over others, beliefs, and behaviors, are noticed and considered more often than other group members (Tankard and Paluck, 2016). Community norms are a dynamic and evolving process that are shaped by: (1) group members' individual and collective behaviors, (2) information about the beliefs and opinions of the referent group (majority group), and (3) signals from institutions that govern, educate or organize the community and which are salient to them (e.g., courts, religion, councils, etc.) (Tankard and Paluck, 2016). Various studies have shown that people tend to align their behavior with social norms. Social norms are produced through beliefs of what is perceived as “desirable” in their community and fear of deviating too far from the norm (Jetten and Hornsey, 2014). Sources of these norms are the opinions and behaviors of social referents (e.g., religious leaders, politicians, activists) who can influence what is acceptable and unacceptable (Boyer, 2022; Chitando and Van Klinken, 2016; Tankard and Paluck, 2016). Social interaction and culture play a huge role in informing an individual's behavior. Prior research in Rwanda has indicated that knowing LGBTQ+ people reduces stigmatizing attitudes (Stojanovski et al., 2024), which may offer insights for intervention.

A community's beliefs about LGBTQ+ people are influenced and shaped by cultural, religious, and societal norms, which are passed down from generation to generation, and community members are expected to adhere to these standards without conscious awareness. African governments present LGBTQ+ rights as Western and un-African, which itself is rooted in categories formulated by colonialists. The legal basis for LGBTQ+ rights is overall negative and hostile toward LGBTQ+ people in much of the African continent (ILGA, 2024). However, community norms are shifting, both positively and negatively, often through the signaling of powerful institutions, such as the courts, legislature, or religious institutions. Uganda recently criminalized the existence of LGBTQ+ by codifying into law lifelong prison sentences for same-sex behavior and death penalties for “aggravated homosexuality,” or repeated same sex acts, amongst others. Ghana, Liberia, and Togo have similarly followed suit by attempting to criminalize LGBTQ+ persons. Such examples showcase how the countries are utilizing institutional signals (laws) to posit that being LGBTQ+ is “unnatural.” Conversely, in Namibia, the Supreme Court found it unconstitutional to criminalize same-sex acts. The changing policies and environments requires continued research to monitor the situation of LGBTQ+ rights.

The results also indicated that older participants are more likely to have discriminatory beliefs compared to younger respondents. This is in line with prior work that shows that as age increases, LGBTQ+ discriminatory beliefs increase (Becker and Scheufele, 2011). A higher level of education was associated with less discriminatory beliefs about LGBTQ+ people. Studies have shown associated reductions in LGBTQ+ discrimination as the level of education increases (Crehan et al., 2021; Schott et al., 2009). Employment status was also associated with shaping discrimination beliefs, as the results show a correlation between employment status and LGBTQ+ acceptance. Employers' perceptions tend to be influenced by various workplace policies and inclusion programs that may foster protection for diverse population groups (Crehan et al., 2021). Moreover, those who are self-employed may be more socially isolated in their employment experiences, thus creating even less exposure to LGBTQ+ and other population groups. These findings underscore the ongoing challenges faced by LGBTQ+ individuals and highlight the need for sensitization and education in various societal contexts in Rwanda. Values Clarification and Attitudes Transformation can play a significant role in shaping a community's perceptions toward LGBTQ+ (Turner et al., 2018). Furthermore, social norms interventions like positively framed messages, contacts with LGBTQ+ individuals, and involving them in community activities have proven to be essential in changing people's values, beliefs, and behavior toward LGBTQ+ individuals (Findor et al., 2023; Yamin et al., 2019).

## 5 Limitations

There are some limitations to note as with any study. First, this was a convenience sample and thus the attitudes represented may not be generalized to all of Rwanda. Given the predominance of urban and higher-educated participants, findings may not generalize to rural or less-educated populations. The study was also cross-sectional, and thus, establishing a causal relationship between the beliefs and the stigma toward the LGBTQ+ community and non-discrimination beliefs is a challenge and reverse causation may be present. Additionally, some terminology used to describe LGBTQ+ issues might not be universally understood, which might carry different connotations to the local language, leading to inaccurate responses from the respondents. Also, the two-question variable of being born LGBTQ+ had a low alpha, and thus, different ways to ask that question in diverse cultural contexts such as Rwanda may be needed to avoid misunderstandings. Response bias is also a concern given that the surveys were conducted in person, and people might have reported less discriminatory attitudes given social desirability.

## 6 Conclusion

The findings from the study conclude that even though many people in Rwanda hold non-discrimination beliefs about LGBTQ+, the acceptance of LGBTQ+ people remains low. These findings suggest associations between beliefs, but further experimental or longitudinal studies are needed to determine causality. Sensitization and educational efforts remain essential.

## Data Availability

Data for this article is not freely available given the sensitive nature, but is available upon request.

## Footnotes

^1^Religious beliefs.pdf
